# Mobile Romberg test assessment (mRomberg)

**DOI:** 10.1186/1756-0500-7-640

**Published:** 2014-09-12

**Authors:** Alejandro Galán-Mercant, Antonio I Cuesta-Vargas

**Affiliations:** Departamento de Psiquiatría y Fisioterapia, Facultad de Ciencias de la Salud, Universidad de Málaga, Andalucia Tech, Cátedra de Fisioterapia y Discapacidad, Instituto de Biomedicina de Málaga (IBIMA), Grupo de Clinimetria (AE-14) Málaga, Malaga, Spain; School of Clinical Sciences of the Faculty of Health at the Queensland University of Technology, Brisbane, Australia

**Keywords:** Frail syndrome, Romberg test, iPhone, Inertial sensor

## Abstract

**Background:**

The diagnosis of frailty is based on physical impairments and clinicians have indicated that early detection is one of the most effective methods for reducing the severity of physical frailty. Maybe, an alternative to the classical diagnosis could be the instrumentalization of classical functional testing, as Romberg test or Timed Get Up and Go Test. The aim of this study was (I) to measure and describe the magnitude of accelerometry values in the Romberg test in two groups of frail and non-frail elderly people through instrumentation with the *iPhone 4*^*®*^, (II) to analyse the performances and differences between the study groups, and (III) to analyse the performances and differences within study groups to characterise accelerometer responses to increasingly difficult challenges to balance.

**Methods:**

This is a cross-sectional study of 18 subjects over 70 years old, 9 frail subjects and 9 non-frail subjects. The non-parametric Mann–Whitney U test was used for between-group comparisons in means values derived from different tasks. The Wilcoxon Signed-Rank test was used to analyse differences between different variants of the test in both independent study groups.

**Results:**

The highest difference between groups was found in the accelerometer values with eyes closed and feet parallel: maximum peak acceleration in the lateral axis (*p* < 0.01), minimum peak acceleration in the lateral axis (*p* < 0.01) and minimum peak acceleration from the resultant vector (*p* < 0.01). Subjects with eyes open and feet parallel, greatest differences found between the groups were in the maximum peak acceleration in the lateral axis (*p* < 0.01), minimum peak acceleration in the lateral axis (*p* < 0.01) and minimum peak acceleration from the resultant vector (*p* < 0.001). With eyes closed and feet in tandem, the greatest differences found between the groups were in the minimum peak acceleration in the lateral axis (*p* < 0.01).

**Conclusions:**

The accelerometer fitted in the *iPhone 4*^*®*^ is able to study and analyse the kinematics of the Romberg test between frail and non-frail elderly people. In addition, the results indicate that the accelerometry values also were significantly different between the frail and non-frail groups, and that values from the accelerometer accelerometer increased as the test was made more complicated.

## Background

Frailty is multidimensional, heterogeneous and unstable, thus distinguishing it from disability or ageing alone [1]. Frail individuals are at particular risk for poor outcomes such as disability, fall, death and hospitalization from minor stressors [2–4]. The diagnosis of frailty is based on several health domains, including physical impairments (e.g., low gait velocity, fatigue and low grip strength), weight loss, and low physical activity [5, 6]. Despite some vagueness in its definition, clinicians have indicated that early detection is one of the most effective methods for reducing the severity of physical frailty and for improving a patient’s well-being. Functional ability assessments aim to detect mobility impairments such as physical weakness so that early interventions are possible [7].

The Romberg test is an appropriate tool to diagnose sensory ataxia, a gait disturbance caused by abnormal proprioception involving information about the location of the joints. It is also proven to be a sensitive and accurate means of measuring the degree of disequilibrium caused by central vertigo, peripheral vertigo and head trauma. The Romberg test is used for the clinical assessment of patients with disequilibrium or ataxia from sensory and motor disorders [8]. The Romberg test, similar to tests such as the 5-stands test and the timed up and go test (TUG), is able to differentiate between subjects with different functional levels [7]. The Romberg test demonstrates loss of postural control in the absence of visual input suggestive of proprioceptive deficit in the lower limbs [9]. When the patient sways or falls with eyes closed while standing with feet together, it is considered to be positive. A positive Romberg’s test has been linked to all causes of proprioceptive deficits, including myelopathies of many causes, tabes dorsalis and sensory neuropathies [10].

The new generation of smartphones often include accelerometers that can detect acceleration [11]. The applications developed for these new electronics can offer read, stored, transferred and displayed kinematic values from the accelerometer [12–16]. These applications evaluate and assess kinematic variables related to gait [17], measuring Cobb angles in x-rays, or provide an objective method to classify levels of physical activity and give an indication of the degree of functional capacity and quality of life [11, 18].

The aim of this study was (I) to measure and describe the magnitude of accelerometry values in the Romberg test in two groups of frail and non-frail elderly people over 70 years old through instrumentation with the *iPhone 4*^*®*^, (II) to analyse the performances and differences between the study groups (frail and non-frail), and (III) to analyse the performances and differences within study groups to characterise accelerometer responses to increasingly difficult challenges to balance.

## Methods

### Design and participants

The study was conducted on a population of older patients over 70 years old (n = 18, 9 frail and 9 non-frail), with a mean age of 79.94 (SD ± 6.14) years, mean weight 60.5 (SD ± 8.44) kilograms, mean height 1.57 (SD ± 0.07) meters and mean body mass index 24.41 (SD ± 2.83). Details of the study were explained to the subjects and consent forms were signed as approved by the ethics committee of the Faculty of Medicine at the University of Malaga, Spain. The participants were classified between frail or non-frail according to the Fried criteria (unintentional weight loss, self-reported exhaustion, weakness, slow walking speed and low physical activity) [5]. The inclusion criteria were anyone over 70 years old who have not presented any of the exclusion criteria. Also, past medical history and falls history over the previous year were also obtained: no history of pain in the last twenty-four hours, previous surgery, presence of a tumour and musculoskeletal disorders in the upper or lower extremity. Patients who needed assistance to mobilise or could not follow simple commands and those with recent neurological impairment were excluded from the study. All participants were clinically examined by a physiotherapist, and no exclusion criteria were identified. Table 1 shows the characteristics of the sample.

**Table 1:** **Anthropometrics and acceleration-based outcomes measures of the Romberg test (** ***N*** **= 18)**

	Frail (***N*** = 9)		Non-frail (***N*** = 9)	
	Mean	SD	Mean	SD
Age (years)	82.78	6.85	77.11	3.89
Weight (kilograms)	55.56	5.48	65.44	8.16
Height (m)	1.56	0.08	1.59	0.08
BMI (kg/m^2^)	23.09	3.11	25.73	1.88
Max_xAccel_EC_para (m/s^2^)	0.08	0.07	0.04	0.03
Min_xAccel_EC_para (m/s^2^)	-0.09	0.13	-0.01	0.04
Max_zAccel_EC_para (m/s^2^)	-0.04	0.19	-0.41	0.15
Min_zAccel_EC_para (m/s^2^)	-0.22	0.19	-0.51	0.17
Max_RV_Accel_EC_para (m/s^2^)	1.09	0.10	1.04	0.01
Min_RV_Accel_EC_para (m/s^2^)	0.92	0.06	0.99	0.01
Max_xAccel_EO_para (m/s^2^)	0.07	0.05	0.03	0.04
Min_xAccel_EO_para (m/s^2^)	-0.06	0.06	-0.02	0.04
Max_zAccel_EO_para (m/s^2^)	0.05	0.18	-0.42	0.15
Min_zAccel_EO_para (m/s^2^)	-0.21	0.19	-0.50	0.16
Max_RV_Accel_EO_para (m/s^2^)	1.10	0.10	1.04	0.01
Min_RV_Accel_EO_para (m/s^2^)	0.93	0.04	0.99	0.01
Max_xAccel_EC_Tandem (m/s^2^)	0.13	0.04	0.18	0.25
Min_xAccel_EC_Tandem (m/s^2^)	-0.13	0.12	-0.09	0.12
Max_zAccel_EC_Tandem (m/s^2^)	0.06	0.28	-0.27	0.28
Min_zAccel_EC_Tandem (m/s^2^)	-0.15	0.23	-0.50	0.18
Max_RV_Accel_EC_Tandem (m/s^2^)	1.10	0.04	1.14	0.14
Min_RV_Accel_EC_Tandem (m/s^2^)	0.91	0.05	0.91	0.12
Max_xAccel_EO_Tandem (m/s^2^)	0.13	0.08	0.08	0.02
Min_xAccel_EO_Tandem (m/s^2^)	-0.05	0.11	-0.04	0.06
Max_zAccel_EO_Tandem (m/s^2^)	0.05	0.26	-0.36	0.20
Min_zAccel_EO_Tandem (m/s^2^)	-0.17	0.25	-0.47	0.19
Max_RV_Accel_EO_Tandem (m/s^2^)	1.06	0.04	1.07	0.02
Min_RV_Accel_EO_Tandem (m/s^2^)	0.94	0.03	0.97	0.03

Max. maximum; Min. minimum; x. x axis; z. z axis; RV. resultant vector; accel. acceleration; EC. eyes closed; EO. eyes open; m. meters; s. seconds; kg. kilograms.

### Test protocol

A pilot study was developed previously in five healthy patients using some test conditions to assess suitability and safe conditions of different functional tests. These data were not included in the final study. Based on this pilot study it was decided to measure the sternum accelerations using:

-  Romberg test with eyes open with feet parallel.
-  Romberg test with eyes closed with feet parallel.
-  Romberg test with eyes open with feet in tandem.
-  Romberg test with eyes closed with feet in tandem.

The functional tasks used in this study provided a range of postural stability challenges suitable for the clinical population of interest (frail and non-frail), but were safe and efficient to administer in a clinical environment. Each task was performed once in normal footwear.

### Definition of frailty

All five characteristics from the original phenotype were retained for the present study [5]: Weight loss was defined as unintentional weight loss of 3 kg or more in the previous three months [5]. Fatigue/exhaustion was defined by a positive answer to the following question: “In the last two weeks have you suffered from… unwillingness to do things or lack of energy? Or fatigue or tiredness?” [5]. Slowness was measured over a 4.57-meter track starting from a standing still position. The slow gait speed criterion was defined as present if the measured gait speed was below the gender and height specific cut-points proposed in the original description of the frailty phenotype [5]. Weakness was defined as the lowest quintile of maximum strength on the dominant hand. The handgrip test is a standardized method for assessing strength of the hand and forearm muscles, The handgrip dynamometer have being found to be highly reliable (ICC = 0.98) and valid (ICC = 0.99) for measuring handgrip strength [19]. The presence of the poor muscle strength criterion was considered as present if below the originally defined thresholds adjusted for gender and body mass index [5]. Low physical activity was was considered as present if the physical activity level fell below the gender-specific thresholds (i.e. <383 kcal/week in men, <270 kcal/week in women) originally proposed by Fried and colleagues [5]. The participants were defined as frail or non-frail according to the presence of ≥3 factors. Others sub-phenotypes as pre-fragile, robust fragile not have been considered for this study.

### Smartphone and the accelerometer

Apple^®^ uses a trialxial gyroscope, accelerometer and a magnetometer in the *iPhone 4*^®^ smartphone [20]. In this study, the app used to record inertial sensor data was *xSensor*^*®*^*Pro, Crossbow Technology, Inc.* This app was available in the *AppStore*^®^. A LIS302DL accelerometer sampling at a frequency of 32 Hz and embedded storage of 20 MB were used in this study in the *iPhone 4*^®^ inertial sensor. A previous study showed that the cell pone (iPhone) accelerometer was accurate and precise compared to a gold standard, with an intra-class correlation coefficient (r^2^ > 0.98). The cell phone accelerometer showed excellent sequential increases with increased in walking velocity and energy expenditure (r^2^ > 0.9). An accelerometer embedded into a cell phone was accurate and reliable in measuring and quantifying physical activity in the laboratory setting manoha [21].

### Data collection and procedures

The smartphone, mounted on the sternum via an elastic neoprene belt, was used to measure body acceleration, estimate both anterior-posterior and medial-lateral body trunk accelerations. Previous studies shows that the essential spatio-temporal trunk characteristics can be obtained by simple trunk accelerometry [22]. The instrumental evaluation in the Romberg test consisted of eyes open (EO) and eyes closed (EC) with feet parallel and feet in tandem. The tests was performed on all subjects with the *iPhone 4*^®^ mounted on the sternum. Study subjects were asked to stand up, with their feet placed so as to maintain the heels together and a 30 degree angle between the right and left toes, and to relax the arms along the body [23]. To ensure similar angles between the feet throughout the test, a guide was made of 2.5 cm green tape on the floor, and the subjects lined their feet up along both arms of the foot-guide. Once the subjects assumed the correct posture, they were asked to maintain the upright standing position for 60 s with their EO and then to maintain the same position for 60 s with their EC [24]; the test was repeated with feet parallel and feet in tandem.

### Data processing and statistical analysis

A computerized automatic analysis was developed to obtain directional acceleration values for further statistical analysis and was performed using basic software package *R*^®^. The variables (directional acceleration value) from the accelerometer were: maximum peak, minimum peak, means and SDs of accelerations in two axes of movements (*x* and *z*). In this study individual acceleration on the vertical axis (*y* axis) was not considered. Furthermore, maximum peak, minimum peak, means and SDs of the resultant vector (RV) accelerations (RV = √ *x*2 + *y*2 + *z*2) were obtained.

Analysis was performed with SPSS version 15 for Windows. Previous analyses were performed to ensure that acceleration values did satisfy the assumptions of normality and linearity. The Kolmogorov-Smirnov test was used as determined by the variables normality of distribution. The non-parametric Mann–Whitney U test was used for between-group comparisons in means values derived from different tasks (EO, EC, tandem feet and parallel feet). The Wilcoxon Signed-Rank test was used to analyse differences between different variants of the test (EO versus EC, tandem feet versus parallel feet) in both independent study groups (frail and non-frail). Statistical results were assumed to be significant at the level of *p* < 0.05.

## Results

Table 1 summarizes the acceleration-based outcome measures of the Romberg test (n = 18).

Table 2 summarizes the Mann–Whitney U test results comparing between-group anthropometric and acceleration values. The highest difference between groups (frail and non-frail) was in the Body Mass Index (*p* < 0.05). In the accelerometer values the greatest differences found between the groups, with EC and feet parallel, were in the maximum peak acceleration in the lateral axis (*p* < 0.01), minimum peak acceleration in the lateral axis (*p* < 0.01) and minimum peak acceleration from the RV (*p* < 0.01). With EO and feet parallel, the greatest differences found between the groups were in the maximum peak acceleration in the lateral axis (*p* < 0.01), minimum peak acceleration in the lateral axis (*p* < 0.01) and minimum peak acceleration from the RV (*p* < 0.001). With EC and feet in tandem, the greatest differences found between the groups were in the minimum peak acceleration in the lateral axis (*p* < 0.01). Finally, the greatest difference found between the groups, with EO and feet in tandem, was in the maximum peak acceleration in the lateral axis (*p* < 0.05).

**Table 2:** **Mann–Whitney U test results comparing between-group accelerations values (** ***N*** **= 18)**

	Percentiles			U	Sig. (2-tailed)
	25%	50%	75%		
Age (years)	74.75	78.0	85.25	23.00	0.120
Weight (kilograms)	54.0	59.0	66.0	23.50	0.130
Height (meters)	1.50	1.57	1.61	32.50	0.479
BMI (kg/m^2^)	22.30	24.77	26.74	18.00	0.047
Max_zAccel_EC_parallel (m/s^2^)	-0.453	-0.235	-0.068	6.00	0.002
Min_zAccel_EC_parallel (m/s^2^)	-0.543	-0.345	-0.230	9.00	0.005
Min_RV_Accel_EC_parallel (m/s^2^)	0.942	0.982	0.993	2.00	0.001
Min_xAccel_EO_parallel (m/s^2^)	-0.075	0-.025	0.013	21.50	0.091
Max_zAccel_EO_parallel (m/s^2^)	-0.435	-0.165	0.098	3.00	0.001
Min_zAccel_EO_parallel (m/s^2^)	-0.558	-0.405	-0.203	10.50	0.008
Max_RV_Accel_EO_parallel (m/s^2^)	1.030	1.040	1.061	21.00	0.085
Min_RV_Accel_EO_parallel (m/s^2^)	0.943	0.973	0.997	0.00	<0.001

Grouping Variable: Frailty. Max. maximum; Min. minimum; x. x axis; z. z axis; RV. resultant vector; accel. acceleration; EC. eyes closed; EO. eyes open; m. meters; s. seconds; kg. kilograms; U. Mann–Whitney U.

Table 3 summarizes the Wilcoxon Signed-Rank test results comparing differences in means values derived from differences between EO versus EC. From the comparison between EO and EC, the highest difference was in the maximum peak acceleration from the RV with feet in tandem in the frail older adults group (*p* < 0.05) (see Table 3).

**Table 3:** **Wilcoxon’s Signed Ranks Test results comparing differences means values derived from differences between EO vs. EC (** ***N*** **= 18)**

	Frail (***N*** = 9)		Non-frail (***N*** = 9)	
	Z	Sig	Z	Sig
Max_xAccel_EO_para (m/s^2^)	-0.070	0.944	-1.265	0.206
Max_xAccel_EC_para (m/s^2^)				
Min_xAccel_EO_para (m/s^2^)	-0.674	0.500	-1.897	0.058
Min_xAccel_EC_para (m/s^2^)				
Max_zAccel_EO_para (m/s^2^)	-0.841	0.400	-0.656	0.512
Max_zAccel_EC_para (m/s^2^)				
Min_zAccel_EO_para (m/s^2^)	-0.593	0.553	-0.282	0.778
Min_zAccel_EC_para (m/s^2^)				
Max_RV_Accel_EO_para (m/s^2^)	-0.700	0.484	-0.889	0.374
Max_RV_Accel_EC_para (m/s^2^)				
Min_RV_Accel_EO_para (m/s^2^)	-0.420	0.674	-0.770	0.441
Min_RV_Accel_EC_para (m/s^2^)				
Max_xAccel_EO_Tandem (m/s^2^)	0.000	1.000	-1.123	0.261
Max_xAccel_EC_Tandem (m/s^2^)				
Min_xAccel_EO_Tandem (m/s^2^)	-1.214	0.225	-1.262	0.207
Min_xAccel_EC_Tandem (m/s^2^)				
Max_zAccel_EO_Tandem (m/s^2^)	-0.365	0.715	-1.836	0.066
Max_zAccel_EC_Tandem (m/s^2^)				
Min_zAccel_EO_Tandem (m/s^2^)	-1.089	0.276	-0.834	0.404
Min_zAccel_EC_Tandem (m/s^2^)				
Max_RV_Accel_EO_Tandem (m/s^2^)	-2.023	0.043	-1.718	0.086
Max_RV_Accel_EC_Tandem (m/s^2^)				
Min_RV_Accel_EC_Tandem (m/s^2^)	-1.214	0.225	-1.362	0.173
Min_RV_Accel_EO_Tandem (m/s^2^)				

Max. maximum; Min. minimum; x. x axis; z. z axis; RV. resultant vector; accel. acceleration; EC. eyes closed; EO. eyes open; m. meters; s. Seconds; para. Parallel.

Table 4 summarizes the Wilcoxon Signed-Rank test results comparing differences in means values derived from differences between feet tandem and feet parallel. The highest difference was found in the non-frail group: maximum peak acceleration from the x axis with EC (*p* < 0.05), minimum peak acceleration from the x axis with EC (*p* < 0.05), maximum peak acceleration from the z axis with EC (*p* < 0.05), maximum peak acceleration from the RV with EC (*p* < 0.01), minimum peak acceleration from the RV with EC (*p* < 0.05), maximum peak acceleration from the x axis with EO (*p* < 0.05), minimum peak acceleration from the x axes with EO (*p* < 0.05), maximum peak acceleration from the z axis with EO (*p* < 0.05), minimum peak acceleration from the z axis with EO (*p* < 0.01), maximum peak acceleration from the RV with EO (*p* < 0.05) and minimum peak acceleration from the RV with EO (*p* < 0.05) (see Table 4).

**Table 4:** **Wilcoxon’s Signed Ranks Test results comparing within group in means values derived from differences between feet tandem vs. parallel (** ***N*** **= 18)**

	Frail (***N*** = 9)		Non-frail (***N*** = 9)	
	Z	Sig	Z	Sig
Max_xAccel_EC_Tandem (m/s^2^)	-1.753	0.080	-2.018	0.044
Max_xAccel_EC_para (m/s^2^)				
Min_xAccel_EC_Tandem (m/s^2^)	-0.135	0.893	-2.196	0.028
Min_xAccel_EC_para (m/s^2^)				
Max_zAccel_EC_Tandem (m/s^2^)	-0.135	0.893	-2.374	0.018
Max_zAccel_EC_para (m/s^2^)				
Min_zAccel_EC_Tandem (m/s^2^)	-2.032	0.042	-0.701	0.483
Min_zAccel_EC_para (m/s^2^)				
Max_RV_Accel_EC_Tandem (m/s^2^)	-1.214	0.225	-2.666	0.008
Max_RV_Accel_EC_para (m/s^2^)				
Min_RV_Accel_EC_Tandem (m/s^2^)	-1.214	0.225	-2.240	0.025
Min_RV_Accel_EC_para (m/s^2^)				
Max_xAccel_EO_Tandem (m/s^2^)	-1.604	0.109	-2.527	0.012
Max_xAccel_EO_para (m/s^2^)				
Min_xAccel_EO_Tandem (m/s^2^)	-0.813	0.416	-2.117	0.034
Min_xAccel_EO_para (m/s^2^)				
Max_zAccel_EO_Tandem (m/s^2^)	-0.135	0.893	-2.536	0.011
Max_zAccel_EO_para (m/s^2^)				
Min_zAccel_EO_Tandem (m/s^2^)	-0.730	0.465	-1.691	0.091
Min_zAccel_EO_para (m/s^2^)				
Max_RV_Accel_EO_Tandem (m/s^2^)	-1.214	0.225	-2.547	0.011
Max_RV_Accel_EO_para (m/s^2^)				
Min_RV_Accel_EC_Tandem (m/s^2^)	-0.134	0.892	-2.547	0.011
Min_RV_Accel_EO_para (m/s^2^)				

Max. maximum; Min. minimum; x. x axis; z. z axis; RV. resultant vector; accel. acceleration; EC. eyes closed; EO. eyes open; m. meters; s. Seconds. para. Parallel.

## Discussion

To our knowledge there have been no previous studies examining a relationship between accelerometry and the clinical balance Romberg test in an older frail and non-frail population with accelerations obtained from the inertial sensor of an *iPhone 4*^®^ smartphone. Significant differences were found between the groups of elderly persons (frail and non-frail) in the accelerometer variables obtained in the kinematic readings of the trunk during the Romberg test. Significant differences were found between results comparing differences between different variants of the test (EO versus EC, tandem feet versus parallel feet) in both independent study groups (frail and non-frail). As the Romberg test could require specialist training and is open to subjective error, accelerometry could be a viable alternative in the measurement of balance.

From the results, values obtained from accelerometry significantly differentiated between the frail and non-frail older adults. The most significant differences were found in EC and feet parallel (most complicated Romberg test variant), and also were significantly different in the maximum peak acceleration in the lateral axis, minimum peak acceleration in the lateral axis and minimum peak acceleration from the RV. With EO and feet parallel, the greatest differences found between the groups were in the maximum peak acceleration in the lateral axis, minimum peak acceleration in the lateral axis and minimum peak acceleration from the RV. With feet parallel and EC, the greatest differences found between the groups were in the maximum peak acceleration in the lateral axis and minimum peak acceleration in the lateral axis. Finally, the greatest difference found between the groups, with EO and feet in tandem, was in the maximum peak acceleration in the lateral axis. Our findings are in agreement with other studies in which accelerometers were also able to indicate significant differences between different population groups [25–30] and are in accordance with other studies in which accelerometers were also able to indicate significant differences between the same population groups of this study (frail and non-frail), but in different functional balance tests or tasks such as Timed Get Up and Go Extended [14], Sit-to-Stand and Stand-to-Sit transitions [15] or analysing frail older adults during a turn transition [16].

Values from the *iPhone 4*^®^ accelerometer increased as the test was made more complicated (EO versus EC and parallel versus tandem). The accelerometry values also were significantly different between feet tandem versus parallel in the non-frail group, but not significantly different between feet tandem versus parallel in the frail group. From the accelerometry values, no significant differences were found between EO versus EC in the non-frail group or in the frail group. Our findings are aligned with other previous studies that have determined the ability of an accelerometer to differentiate between functional tasks with increasing complexity [25–27, 30, 31]. A previous study [27], aligned with our findings, found that accelerometry was able to significantly differentiate between EO and EC in different conditions. However, unlike the present study, they did not use *iPhone 4*^*®*^ technology to collect kinematic variables and used a different population group (young and elderly healthy subjects). Three previous studies [25, 26, 30], in contrast to the present study, found that the accelerometer variables of both amplitude and frequency identified differences between conditions of EO and EC while standing on a firm surface and on a mat in a population of older fallers. However, unlike the present study, they did not use *iPhone 4*^*®*^ technology to collect kinematic variables and their goal was to examine if a correlation exists between accelerometry-derived variables and the Berg Balance Scale in older adults, and to characterise accelerometer responses to increasingly difficult challenges to balance.

The authors claim that the difference without visual feedback may only represent a marginally different challenge to balance control and that this difference is difficult to detect. However, therapists should be cautious in interpreting performance, because many underlying sensory (visual and vestibular), motor, and orthopedic problems may contribute to instability [32]. Documentation of improvements in ability to organize sensory information or balance can be obtained by periodic reassessment. Analysis of the patterns of instability over the four test or conditions provides therapists with insight into which sense a patient depends on to maintain stability.

The results obtained open up the way for further research in the future, although this study presents a series of limitations: the small subject numbers of sample, not allowed the generalisability of the findings. Men and women have different characteristics, and it would be interesting to analyse differences in the kinematic data by gender in the Romberg test. It would be interesting to consider futures studies in order to analyze the predictive capability of the variables in the Romberg test between the study groups.

## Conclusions

On the basis of these results, we conclude that the accelerometer fitted in the *iPhone 4*^*®*^ is able to study and analyse the kinematics of the Romberg test between frail and non-frail elderly people. In addition, the results indicate that the accelerometry values also are significantly different between the frail and non-frail groups, and that values from the *iPhone 4*^®^ accelerometer increased as the test was made more complicated (feet parallel versus feet tandem). Future studies should aim to analyse the predictive capability of the kinematic variables that showed statistically significant differences in the Romberg test between non-frail and frail elderly persons.
